# Rapid Experimental Evolution of Pesticide Resistance in *C. elegans* Entails No Costs and Affects the Mating System

**DOI:** 10.1371/journal.pone.0003741

**Published:** 2008-11-17

**Authors:** Patricia C. Lopes, Élio Sucena, M. Emília Santos, Sara Magalhães

**Affiliations:** 1 Programa Graduado em Áreas da Biologia Básica e Aplicada (GABBA), Faculdade de Medicina da Universidade do Porto, Porto, Portugal; 2 Centro de Biologia do Desenvolvimento, Instituto Gulbenkian de Ciência, Oeiras, Portugal; 3 Centro de Biologia Ambiental, Faculdade de Ciências da Universidade de Lisboa, Campo Grande, Lisbon; The University of New South Wales, Australia

## Abstract

Pesticide resistance is a major concern in natural populations and a model trait to study adaptation. Despite the importance of this trait, the dynamics of its evolution and of its ecological consequences remain largely unstudied. To fill this gap, we performed experimental evolution with replicated populations of *Caenorhabditis elegans* exposed to the pesticide Levamisole during 20 generations. Exposure to Levamisole resulted in decreased survival, fecundity and male frequency, which declined from 30% to zero. This was not due to differential susceptibility of males. Rather, the drug affected mobility, resulting in fewer encounters, probably leading to reduced outcrossing rates. Adaptation, i.e., increased survival and fecundity, occurred within 10 and 20 generations, respectively. Male frequency also increased by generation 20. Adaptation costs were undetected in the ancestral environment and in presence of Ivermectin, another widely-used pesticide with an opposite physiological effect. Our results demonstrate that pesticide resistance can evolve at an extremely rapid pace. Furthermore, we unravel the effects of behaviour on life-history traits and test the environmental dependence of adaptation costs. This study establishes experimental evolution as a powerful tool to tackle pesticide resistance, and paves the way to further investigations manipulating environmental and/or genetic factors underlying adaptation to pesticides.

## Introduction

Pesticides and antibiotics have been developed to induce high mortality rates on populations of parasites and pests. This imposes a strong selection pressure on these organisms, which may lead to the evolution of resistance to such xenobiotics. Resistance has indeed been observed in an impressive number of organisms [1], [2], [3]. Due to its ubiquity, pesticide resistance is also currently a model trait for the study of adaptation to novel environments [4]. Laboratory experiments with microorganisms and field studies with multicellular organisms have shown that resistance to xenobiotics occurs within short time frames [5], [6], [7], [8].

In addition to causing lethality, xenobiotics may also affect the morphology, life history, or behaviour of organisms without killing them. For example, many pesticides reduce the fecundity and/or longevity of organisms [9], whereas others cause paralysis, thereby compromising the ability of organisms to find food or mates, or to escape from potential predators [10], [11], [12]. Despite being frequently overlooked, these sublethal effects can nonetheless affect the performance of organisms and significantly impact fitness [9], [13]. Hence, it is expected that natural selection will operate towards reducing these deleterious effects induced by pesticides.

A crucial aspect for both resistance management and our understanding of the evolutionary consequences of adaptation is to evaluate whether the evolution of resistance entails a cost in terms of performance in other environments. Indeed, the presence of a cost opens the possibility for managing resistance by creating areas where the pesticide is not spread [14]. From a fundamental perspective, a cost of adaptation has often been evoked as the mechanism underlying the evolution of specialization. Examples from the literature so far suggest that a cost of resistance is indeed common, but that its intensity is variable [15], [16], [17]. The presence of a cost of adapting to a pesticide, as well as its specific evolutionary dynamics will depend on the degree of resemblance among environments [18], [19], on the genetic basis of adaptation [16], on the genetic background of the organism [20], and on the intensity of selection [21], [22], [23].

Experimental evolution in replicate populations exposed to pesticides can contribute to our understanding of the evolutionary fate of lethal and sublethal effects caused by chemical stress, as well as to follow the building up of a cost of resistance. To date, few studies have been carried out on the experimental evolution of pesticide resistance in multicellular organisms using replicated evolving lines, and none involve an androdioeceous organism. In this mating system, males result from an outcrossing event between hermaphrodites and males, whereas hermaphrodites are also able to undergo selfing [24]. One possible sublethal effect of the pesticide is to affect this mating system, and the build-up of resistance may also interact with it.

In this study, we followed the experimental evolution of resistance of the androdioecious free-living nematode *Caenorhabditis elegans* to the widely used pesticide Levamisole. This nematicide targets the nicotinergic acetylcholine receptor, resulting in depolarisation of neuronal and muscle cells [25], [26]. Apart from inducing severe mortality, Levamisole modifies several life-history and behavioural traits of *C. elegans*, including egg laying and mobility [27], [28]. Hence, we measured adaptation not only as changes in life-history traits such as fecundity and survival, but also as behavioural modifications. To investigate whether resistance entailed a cost, we measured the performance of resistant populations in the ancestral environment. As detecting a cost of resistance may depend on the environment where this cost is measured [16], we also measured this cost in the presence of another nematicide, Ivermectin. Ivermectin acts by being an agonist of glutamate-mediated chloride channels, resulting in the hyper-polarization of the membrane of neuronal and muscle cells [29]. Since Levamisole and Ivermectin operate on excitatory and inhibitory networks, respectively, a strong trade-off in adaptation to the two nematicides is expected. Indeed, negative cross-resistance between these pesticides has been shown [30]. Therefore, we used Ivermectin as an environment where the probability of detecting a cost of adapting to Levamisole is expected to be maximized.

## Results

Pesticides significantly affected the survival and fecundity of all populations (GLM, effect of environment, F_2,16.045_ = 44.05; *P*<0.0001 and F_2,16_ = 98.34; *P*<0.0001). The interaction between the environment and the selection regime was significant for survival, but not for fecundity (F_2,16_ = 12.34; *P* = 0.0006 and F_2,16_ = 2.64, *P* = 0.1, respectively). Subsequent analyses were performed on each environment separately.

**Figure 1:**
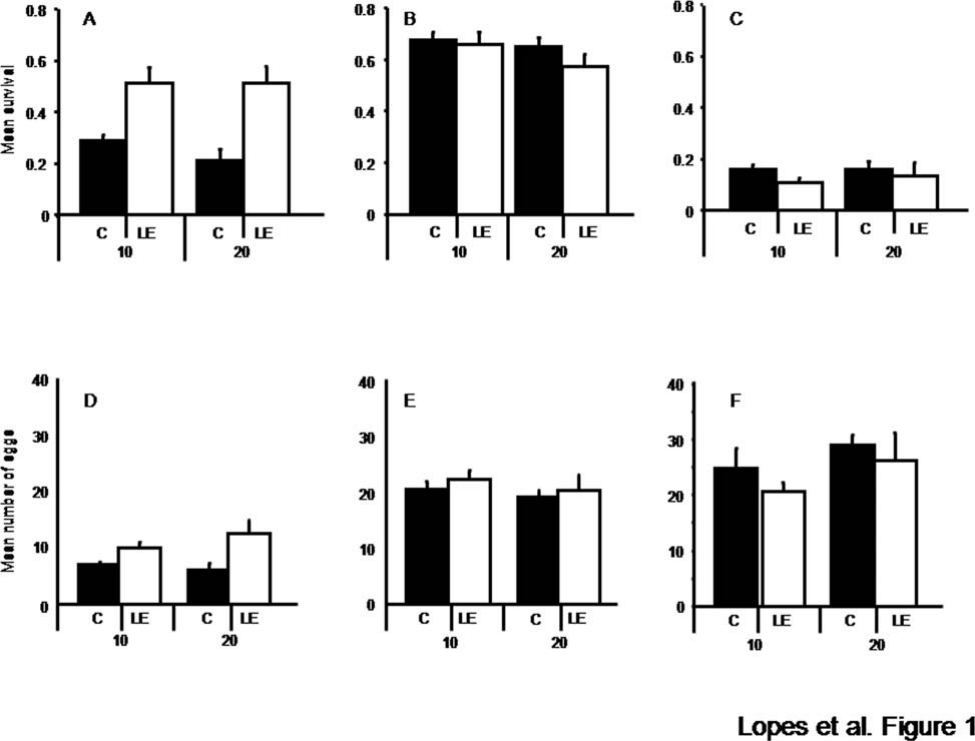
Adaptation and its potential costs. Life history traits of populations in three different environments: Levamisole (a) and (d), Control (b) and (e), and Ivermectin (c) and (f). Survival (a, b, c) was measured as the proportion of individuals surviving from egg to adulthood (after 3 days). Fecundity (d, e, f) was assessed by counting the number of eggs per hermaphrodite after individual bleaching at day 4. Black bars: Control populations; white bars: LE populations. Vertical bars correspond to the standard error of the mean of the five populations in each selection regime.

Populations evolving in Levamisole had higher survival and fecundity in this environment than populations evolving in a Control environment (Fig. 1a and 1d; Table 1, effect of selection regime). Thus, exposure to Levamisole resulted in adaptation to this environment within 20 generations. However, adaptation was very heterogeneous among populations (Table 1, effect of population). Differences in fecundity between selection regimes were observed at generation 20 only, whereas differences in survival were established at generation 10 and remained constant thereafter (Figure 1; Table 1, interaction generation*selection regime).

**Table 1:** Statistical analysis of life-history traits.

Trait	Source	Environment					
		Levamisole		Control		Ivermectin	
		F (d.f.)	*P*	F (d.f.)	*P*	F (d.f.)	*P*
Survival	G	1.59(1)	0.26	2.87(1)	0.094	0.27(1)	0.6
	SR	16.06(1)	**<0.0001**	1.00(1)	0.35	2.14(1)	0.15
	SR (P)	4.83(8)	**<0.0001**	1.94(8)	0.064	1.0.1(8)	0.49
	G*SR	NS	NS	NS	NS	NS	NS
	G*SR(P)	NS	NS	NS	NS	NS	NS
Fecundity	G	0.12(1)	0.74	1.07(1)	0.32	2.57(1)	0.13
	SR	11.69(1)	**0.009**	0.32(1)	0.58	1.52(1)	0.23
	SR (P)	6.65(8)	**0.007**	1.6(8)	0.26	1.4(8)	0.32
	G*SR	7.03(1)	**0.024**	NS	NS	NS	NS
	G*SR(P)	1.83(8)	0.069	6.59(8)	**<0.0001**	8.2(8)	**<0.0001**

G: Generation; SR: Selection Regime; SR(P): Population nested within Selection Regime; F: F value; d.f.: degrees of freedom; *P*: significance. Survival: number of individuals reaching adulthood; Fecundity: number of eggs carried by hermaphrodites at day 4. Non-significant interactions (*P<*0.1, “NS”) were removed from the model. *P*<0.05 are highlighted in bold.

In the Control environment, survival and fecundity of individuals from LE populations was not significantly different from that of individuals from C populations (Fig. 1b and 1e; Table 1, effect of selection regime). Thus, adaptation to Levamisole entailed no cost in the ancestral environment. These traits did not differ between generations (Table 1, effect of generation), but fecundity in some populations changed between generations, resulting in a significant interaction between generation and population (Table 1). In Ivermectin, the survival and fecundity of the LE populations did not differ significantly from that of C populations (Fig. 1c and 1f; Table 1, effect of selection regime). Therefore, resistance to Levamisole was not accompanied by a cost in an environment with Ivermectin. As in the Control environment, a significant interaction between generation and population was found (Table 1).

**Figure 2:**
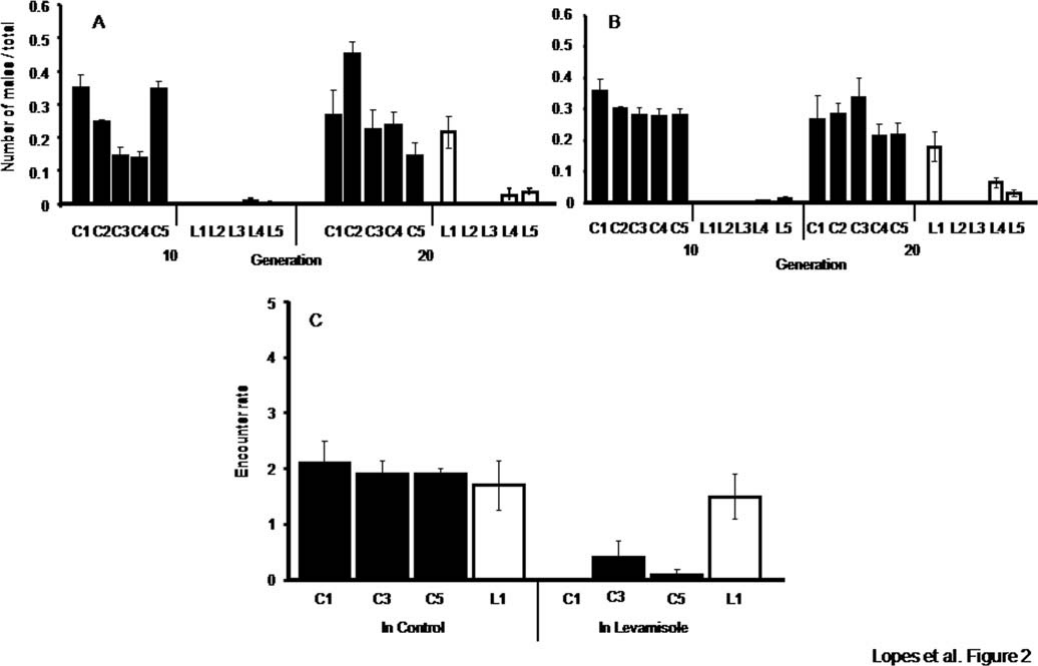
Evolution of the mating system. Male frequency (number of males/total number of individuals) of all populations in the environment with Levamisole (a) and in the Control environment (b), measured at day 3. (c): behavioural observations of the populations C1, C3, C5 and L1 in the Control or in the Levamisole environment during 20 minutes: encounter rates between males and hermaphrodites: Black bars: Control populations (C1–C5); white bars: LE populations (L1–L5). Vertical bars correspond to the standard error of the mean.

Male frequency did not differ significantly between the Levamisole and the Control environment (Fig. 2; GLM, effect of the environment, F_1,8_ = 0.8, *P* = 0.39). In addition, no significant interaction was found between the environment and the population, selection regime nor generation (GLM, *P*>0.3 for all interactions). Therefore, the environment where individuals developed did not significantly affect the male frequency observed. Male frequency differed significantly between LE and C populations when exposed to Levamisole (Fig. 2a; GLM, effect of the selection regime: F_1,8_ = 34.79, *P*<0.0001). Indeed, the male frequency of LE populations at generation 10 was near 0%, whereas that of C populations varied between 14 and 35%. By generation 20, male frequency increased in 3 of the 5 LE populations (Fig. 2a, GLM. interaction generation*population(selection regime), F_9,80_ = 3.51, *P* = 0.0005).

To understand the disappearance of males after 10 generations in Levamisole, we tested the effect of this drug on the survival of each sex separately. Significant differences in susceptibility were found between sexes (F_1,40_ = 68.48; *P* = 0.001). However, males were less sensitive to Levamisole than hermaphrodites. Indeed, on average 43.9±3.42% of the hermaphrodites of each population survived to Levamisole, while this proportion was of 69.3±1.4% for males (on average 97.7±1.4% of the hermaphrodites and 100% of the males survived in the control). Hence, differences in susceptibility to the pesticide between sexes do not explain the disappearance of males in the LE populations. Subsequently, we tested if outcrossing was impaired in the Levamisole environment.,In populations naïve to the Levamisole environment (the C populations), the number of encounters in Levamisole is significantly lower than in the Control environment (Fig. 2c; F_1,4_ = 23.28; *P* = 0.017). This is not the case for the LE1 population, for which these variables do not differ across environments (Fig. 2c; t_18_ = 0.33; *P* = 0.74). The rate of encounter of LE1 individuals in the Control environment is comparable to that of C individuals (Fig. 2c, t_11_ = 2.2; *P* = 0.58), and so is the male frequency of that population (Fig. 2a,b). However, compared to C individuals, individuals of the LE1 population encounter mates more often in the Levamisole environment (Fig. 2c; t_10_ = 2.23; *P* = 0.006). Therefore, resistance to Levamisole translated also into a behavioural change of the individuals, which allowed for an increase in male frequency.

## Discussion

Experimental evolution of *C. elegans* populations in a Levamisole-enriched environment resulted in adaptation to this environment within 20 generations. This adaptation to a novel environment entailed no cost in the ancestral environment or in Ivermectin, another pesticide with an opposite physiological mode of action. Levamisole paralyzed the nematodes. This resulted in fewer encounter rates between males and hermaphrodites and led to the disappearance of males from the populations. A build-up of resistance has re-established the mobility of the worms, and concomitantly the male frequency increased.

Resistance in our outbred populations accumulated within very few generations. Therefore, adaptation was most likely due to the standing genetic variation of populations. The fact that pesticide resistance is a trait that is relatively easy to select for under artificial selection [31] is in agreement with the prediction that genes conferring pesticide resistance may be present in populations at low frequencies. Even though data from natural populations and from artificial selection suggest that resistance can indeed rapidly accumulate, this is the first study providing a direct demonstration of the speed of this process. The speed of adaptation varied with the trait measured. Indeed, survival increased within 10 generation and had reached a plateau at 20 generations, whereas fecundity increased mostly at generation 20. This difference suggests that these traits evolve independently, at least to a certain extent.

The rapid evolution of resistance to Levamisole was not accompanied by a cost in the ancestral environment. This result differs from most studies of pesticide resistance, where a cost was detected [16], [32], [33], [but see 34]. This discrepancy may be due to the fact that we used a selection pressure that allowed the survival of 25% of the initial population, whereas most studies deal with natural populations, where pesticide doses aim at eradicating all individuals of a pest population. In those cases, probably only the most effective mutation conferring resistance is selected. Indeed, most resistance mutations described are a one-base-pair change that modifies the binding site of the pesticide in the corresponding neuroreceptor, which is likely to be costly, as other molecules also bind to that site [2]. As the size of populations surviving pesticide use increases, several gene combinations may build up and be selected, hence reducing the probability of a costly resistance.

Detecting a cost of resistance may depend on the environment where such cost is measured [18], [19]. With the aim of maximizing the possibility of detecting a cost, we selected an environment expected to have an opposite physiological effect on the worms to that imposed by Levamisole. As Levamisole and Ivermectin operate on excitatory and inhibitory circuits respectively, resistance to one of these drugs may well increase the susceptibility to the other, entailing a cost of adaptation. However, even in such an environment, resistance to Levamisole did not entail any cost. Therefore, the lack of cost is probably not contingent on the environment where costs were tested. It is possible that the period of experimental evolution was too short to create a measurable cost of adaptation. However, the fact that adaptation was detected during the experimental period, and that it was not accompanied by a cost indicates that adaptation to each environment is, at least to a certain extent, determined by independent loci [35], [36].

Exposure to Levamisole resulted in fewer encounters between males and hermaphrodites. Since males are produced mainly as the result of an outcrossing event, which involves an encounter between a male and a hermaphrodite, males in populations exposed to the pesticide disappeared within 10 generations. This result supports the hypothesis that encounter rates are an important factor in determining male frequency in *C. elegans* populations [37], [38], and may underlie the frequencies in the base population. However, in other studies of experimental evolution in the laboratory, where encounter rates were probably similar as those of our base population, male frequencies were extremely low [39], [40]. Hence, additional factors need to be invoked to explain the male frequency observed in the base population used in this study. Had we used a non-selfing species, Levamisole would probably have impaired nearly all mating events, leading to severe reduction in population growth. This suggests that sublethal pesticide effects can have dramatic consequences on populations. As *C. elegans* is capable of both selfing and outcrossing, the action of the pesticide resulted in a remarkable reduction in outcrossing rates, but populations were maintained through selfing.

If the speed at which males disappeared from the populations exposed to Levamisole was striking, the same is true for the pace at which male frequency increased in the populations that became resistant to the drug. The latter suggests that outcrossing is indeed advantageous in these populations; otherwise male frequency would be expected to remain near 0% [40]. 30% of males is a frequency that corresponds to the locally-stable equilibrium predicted by Stewart and Phillips (2002) [40]. This male frequency may be expected because outcrossing produces two to four times the offspring obtained through selfing [41]. Therefore, the increase in male frequency observed, as a result of restored mobility, may be seen as yet another expression of the evolution of pesticide resistance in these populations.

Pesticide resistance has been used as a ‘model trait’ to study adaptation to novel environments for the past 20 years [4], [31]. Our study underscores the potential use of this model trait in experimental evolution. By using pesticide resistance in a controlled setting, we were able to shed light on the reciprocal interactions between behavior and evolution, as well as to test the multidimensionality of adaptation costs. However, the potentialities of this system are not restricted to the results obtained in the current study. Using experimental evolution to tackle pesticide resistance allows for the manipulation of a variety of environmental and genetic factors. Indeed, manipulating selection intensity, environmental stability, population size and genetic background, provide direct tests of the effects such factors may have on the process and outcome of adaptation.

## Methods

The base population of Caenorhabditis elegans used in this study was composed of a mixture of the strains used in Teotónio et al. 2006 [42]. It was kept in the experimental conditions described in Manoel et al. 2007 [43], for over 80 generations prior to our study. Levamisole (Levamisol hydrochloride, C11H12N2S · HCl), an imidazothiazole and Ivermectin (22,23-Dihydroavermectin B1), a macrocyclic lactone, were purchased from Sigma-Aldrich.

From the initial population, we derived 10 experimental lines: five maintained in standard conditions [C1–C5] and five kept in plates containing the nematicide Levamisole (LE1–LE5). The populations were cultured for 20 generations at 20°C and 80% RH and frozen at generation 10 (G10) and 20 (G20) for later use in the assays. Our standard experimental evolution protocol followed that of Manoel *et al.* 2007 [43]. Each generation lasted 4 days. At day 1, 1000 individuals at the first larval stage (L1) were placed onto Petri dishes (9 cm diameter) containing Nematode Growth Media-light agar (NGM) (US Biological) with a lawn of HT115 *Escherichia coli* as food source, then incubated for 3 days. At day 4, individuals were washed off the plates and exposed to a hypochloride/sodium hydroxide solution, which kills all life stages except the eggs inside the hermaphrodites. These eggs were subsequently kept in a M9 buffer solution in 15mL falcon tubes in an incubator at 20°C and 120 rpm overnight. The next day, the number of larvae on each tube was estimated with five sample drops of 5 µL from each tube and the volume corresponding to 1000 of individuals was placed in fresh Petri dishes. Each population was composed of 10 Petri dishes, hence N = 10 000, individuals per population. The NGM-light agar in which LE populations were kept contained Levamisole 0.15 mM. This concentration was lethal for 75% of the individuals in the base population, but had no effect on bacterial growth (T-test, N = 10 petri dishes per environment, t = 1.26, *P* = 0.23).

Adaptation was assessed by comparing the performance of LE populations to those of C populations in petri dishes containing Levamisole (hereafter the Levamisole environment), while the control (drug-free) environment and the environment containing Ivermectin 0.04 µM served to measure potential costs of adaptation. Prior to testing performance, all populations (C1–C5 and LE1–LE5) spent three generations in a drug-free environment, to ensure that the responses observed were due to genetic differences among populations. Subsequently, 100 eggs from each population were placed onto fresh petri dishes of each environment (N = 5 plates/environment) and incubated for 3 days at 20°C and 80% RH. When individuals reached adulthood (4^th^ day of culture), 30 gravid hermaphrodites from each plate were collected and individually submitted to a hypochloride/sodium hydroxide solution. The surviving eggs were counted, yielding the fecundity measure. This method mimics the conditions used in the experimental evolution setup, but at an individual level. The plates with the remaining individuals were placed at 4°C for two days to immobilize the individuals to be counted. Survival was obtained by counting the number of individuals per plate (accounting for the 30 removed to measure fecundity) and dividing it by the initial number of eggs plated (100). Male frequency was estimated as the ratio between the number of males and the total number of individuals counted.

Next, we aimed at understanding the male frequencies observed (cf. Results). We first tested whether males were more susceptible to Levamisole than hermaphrodites. 20 adult males and 20 hermaphrodites from each C population at generation 20 were placed separately in Levamisole and in Control plates (5 plates per population per environment). After one day, the number of individuals surviving was counted. Subsequently, we measured the encounter rate between males and hermaphrodites. Four hermaphrodites from one population were placed on a small drop of bacteria (10 µL) that had grown overnight in a 5-cm diameter plate containing either 0.15 mM Levamisole or no drugs. Subsequently, a male was introduced and this group was observed for 20 minutes. We registered the number of male-hermaphrodite encounters. This was done ten times for C1, C3, C5 and LE1 at generation 20.

Differences in survival and fecundity were first analyzed with General Lineal Models using the GLM procedure in SAS. The factors of the model were “environment” (levamisole, ivermectin or control), “generation” (10 or 20), “selection regime” (LE or C lines), a factor “selection line” (C1–C5 and LE1–LE5) nested to the factor “selection regime”, and the interactions “environment*selection regime”, “generation” * “selection regime”, “environment*selection line” and “generation” * “selection line”. The factor “selection line” and its interactions with other factors were considered random factors. The interaction terms with P-values larger than 0.1), were sequentially dropped from the analysis and included in the error term [44]. Subsequently, we performed statistical tests within each environment to answer specific questions. Adaptation was tested by comparing survival and fecundity of LE and C populations in the Levamisole environment. The analysis and the factors used were the same as before, except for the factor environment and its interactions with the other factors. A cost of adaptation was tested with the same model, but with the data collected in the other two environments.

Differences in male frequencies were tested with a GLM procedure in SAS, with the same model as for fecundity and survival, but excluding the Ivermectin environment. Comparisons between the control and the levamisole environment aimed at testing whether an immediate physiological effect of the environment could affect the male frequencies observed; comparisons among selection regimes tested the effect of the pesticide on male frequency, while comparisons between generations of the levamisole lines tested recovery due to the evolution of resistance. To test differences in survival between males and hermaphrodites, only C populations were used. The sex of the individuals was introduced as a fixed factor and population as a random factor. To test the effect of the Levamisole environment on the ability to find a mate, we compared the number of encounters of individuals from C populations in the Levamisole versus the Control environment. Environment was introduced as a fixed factor and population as a random factor. As there were no significant differences among populations, these were grouped in the subsequent analysis. To test whether resistant individuals had recovered their ability to find a mate, we used individuals from the most resistant population at generation 20, LE1, and compared their behavior to that of individuals from the C selection regime. To test whether the encounter rates of individuals from the LE1 population varied between environments, we performed a T-test in Microsoft Excel.
